# LMA or vivipary? Wheat grain can germinate precociously during grain maturation under the cool conditions used to induce late maturity alpha-amylase (LMA)

**DOI:** 10.3389/fpls.2023.1156784

**Published:** 2023-06-29

**Authors:** Sarah R. Peery, Scott W. Carle, Matthew Wysock, Michael O. Pumphrey, Camille M. Steber

**Affiliations:** ^1^ Department of Crop and Soil Sciences, Washington State University, Pullman, WA, United States; ^2^ U.S. Department of Agriculture – Agricultural Research Service, Wheat Health, Genetics and Quality Research Unit, Pullman, WA, United States

**Keywords:** vivipary, late maturity alpha-amylase, LMA, preharvest sprouting, *Triticum aestivum*, wheat, germination, dormancy

## Abstract

**Introduction:**

This study found that wheat (*Triticum aestivum*) grain can germinate precociously during the maturation phase of grain development, a phenomenon called vivipary that was associated with alpha-amylase induction. Farmers receive severe discounts for grain with low falling number (FN), an indicator that grain contains sufficiently elevated levels of the starch-digesting enzyme alpha-amylase to pose a risk to end-product quality. High grain alpha-amylase can result from: preharvest sprouting (PHS)/germination when mature wheat is rained on before harvest, or from late maturity alpha-amylase (LMA) when grain experiences cool temperatures during the soft dough stage of grain maturation (Zadoks growth stage 85). An initial LMA-induction experiment found that low FN was associated with premature visible germination, suggesting that cool and humid conditions caused vivipary.

**Methods:**

To examine whether LMA and vivipary are related, controlled environment experiments examined the conditions that induce vivipary, whether LMA could be induced without vivipary, and whether the pattern of alpha-amylase expression during vivipary better resembled PHS or LMA.

**Results:**

Vivipary was induced in the soft to hard dough stages of grain development (Zadok’s stages 83-87) both on agar and after misting of the mother plant. This premature germination was associated with elevated alpha-amylase activity. Vivipary was more strongly induced under the cooler conditions used for LMA-induction (18°C day/7.5°C night) than warmer conditions (25°C day/18°C night). Cool temperatures could induce LMA with little or no visible germination when low humidity was maintained, and susceptibility to vivipary was not always associated with LMA susceptibility in a panel of 8 varieties. Mature grain preharvest sprouting results in much higher alpha-amylase levels at the embryo-end of the kernel. In contrast, vivipary resulted in a more even distribution of alpha-amylase that was reminiscent of LMA.

**Discussion:**

Vivipary can occur in susceptible varieties under moist, cool conditions, and the resulting alpha-amylase activity may result in low FN problems when a farm experiences cool, rainy conditions before the crop is mature. While there are genotypic differences in LMA and vivipary susceptibility, overlapping mechanisms are likely involved since they are similarly controlled by temperature and growth stage, and result in similar patterns of alpha-amylase expression.

## 1 Introduction

This paper examined precocious germination as a cause of elevated α-amylase expression in wheat (*Triticum aestivum L*.). The appropriate regulation of seed germination is critical both to species survival and to successful agriculture because seeds are the main mechanism of plant propagation and often the crop harvested (reviewed by Finkelstein et al., 2008). The seeds of cereals are caryopses, also referred to as grain or kernels. Cereal grains including wheat, barley, rye, and rice can undergo preharvest sprouting, the inappropriate germination of mature grain on the mother plant when rained on before harvest (reviewed by Derera, 1989; Rodriguez et al., 2015). Preharvest sprouting can result in serious losses for farmers in both mild and extreme cases. In extreme cases when seedling emergence is obvious, the grain is unsuitable for human consumption and may be sold as animal feed. However, even before wheat grain is visibly germinated, the induction of the starch-degrading enzyme α-amylase can pose a risk to end-product quality because starch digestion reduces its gelling capacity, leading to fallen cakes and sticky noodles (reviewed in Steber, 2017; Bettge, 2018). Problems with elevated α-amylase are detected using the falling number (FN) method in the wheat industry where a low FN indicates elevated α-amylase (Perten, 1964; Ross and Bettge, 2009). The falling number is the time required in seconds (sec) for a stirrer to fall through a heated flour/water mixture, where more starch digestion by α-amylase results in decreased viscosity and a lower FN. Wheat grain with an FN below 300 sec is considered to have too much α-amylase and an elevated risk of poor end-product quality.

Preharvest sprouting generally results from insufficient grain dormancy at maturity (reviewed by Mares and Mrva, 2014; Rodriguez et al., 2015). Dormant grain cannot germinate under favorable conditions. After pattern formation, seeds enter the maturation phase of seed development during which stored reserves accumulate and the seed acquires both desiccation tolerance and dormancy. The plant hormone abscisic acid (ABA) induces dormancy and desiccation tolerance during seed maturation, preventing premature grain germination. In wheat and other cereals, grains do not normally germinate before completion of the maturation phase of seed development because the hormone ABA is present at high levels (reviewed by Derera, 1989; McCarty, 1995). Dormant cereal grains can acquire the ability to germinate through dry after-ripening, a period of dry storage during which dormancy is lost (Tuttle et al., 2015; Martinez et al., 2016). Such non-dormant, after-ripened grains can remain metabolically quiescent and ungerminated as long as they are dry. Upon imbibing water under favorable lighting and temperature conditions, nondormant grains will germinate and grow. Wheat can also lose dormancy through cold stratification, when imbibing water under cool temperatures. Wheat dormancy loss through both after-ripening and cold stratification is associated with decreasing sensitivity to ABA’s inhibition of germination.

In the tropical cereal maize, caryopses/kernels become metabolically quiescent during seed maturation, but do not achieve dormancy sufficient to prevent germination under favorable moisture conditions. Some genotypes of maize fail to become quiescent during development and can, therefore, germinate on the mother plant before maturity. This premature germination is called vivipary, a reference to live birth. The *viviparous* (*Vp*) mutants of maize proceed directly from embryo development into germination without first completing seed maturation and becoming quiescent (McCarty, 1995; White et al., 2000). Gene cloning showed that the maize *viviparous* mutants result either from loss of ABA hormone biosynthesis or loss of genes needed for response to ABA. Vivipary of maize can also be induced by fungal pathogens such as fusarium (Galindo and Romero, 1983). Here we report an example of vivipary in another cereal, wheat.

The two major causes of elevated α-amylase in wheat grain are preharvest sprouting (PHS) and late maturity α-amylase (LMA) (reviewed by Mares and Mrva, 2014; Sjoberg et al., 2020). During germination, gibberellin A (GA) hormone from the embryo induces α-amylase expression in the scutellum and aleurone cell layer of the wheat grain (Jacobsen et al., 1995; Mrva and Mares, 1996). Because GA from the embryo induces this expression, α-amylase levels are much higher at the embryo-proximal end of the germinating mature grain. This α-amylase is eventually released into the endosperm where it digests long starch chains into saccharides that nourish the germinating seedling. LMA is the induction of α-amylase by cool temperatures during grain development in the soft dough stage of grain maturation (Zadoks growth stage 85; Zadoks et al., 1974) in the absence of rainfall. While both LMA and PHS result in the expression of α-amylase in the aleurone cell layer, α-amylase is expressed randomly throughout the aleurone during LMA resulting in similar levels at the embryo-proximal and distal end of the grain, whereas PHS results in higher levels at the embryo-proximal end (Mrva and Mares, 1996; Mrva et al., 2006). Thus, the location of α-amylase expression can be used to distinguish LMA from PHS. Currently, low FN/elevated α-amylase from LMA is considered a developmental problem, whereas low FN from PHS is considered part of mature grain germination. However, this distinction became less clear when we observed premature germination or vivipary in an LMA induction experiment.

This study was initiated because visible germination was observed in immature grain from a field LMA-induction experiment in which detached wheat spikes were placed in a cool incubator during the grain maturation phase of seed development. LMA induction conditions appeared to trigger premature germination or vivipary in wheat because incubator conditions were cool and humid. This raised several questions including whether vivipary can be reproducibly induced in wheat under cool and humid conditions, whether LMA is actually a form of vivipary, and finally whether the methods used for LMA induction need to be modified to prevent vivipary. To address these questions, we examined when immature wheat grains acquire the ability to germinate either threshed or on the mother plant. We also modified the LMA-induction assay to reduce visible germination of wheat grain.

## 2 Materials and methods

### 2.1 Summary of methods used to assay LMA, vivipary, and preharvest sprouting.

This study employed several approaches to assay three traits, LMA, vivipary, and preharvest sprouting (PHS) of wheat. These methods (A through G) are described below and summarized in **Table 1**.

**Table 1:** Comparison of methods used to assay traits.

	LMA TRAIT	VIVIPARY TRAIT
**Method:** Seed Age: Treatment: Temperature: Seed source: Figures:	**A. Field LMA induction** Immature, detached tillers Low humidity, cool treatment vs warm 18°C day/7.5 °C night vs 25°C day/18°C night Field-grown detached tillers **Figure 1**, **Figure 7**, **Figure 8**	**B. Field Vivipary Induction** Immature, detached tillers High humidity, cool treatment vs warm 18°C day/7.5 °C night vs warm outdoor Field-grown detached tillers **Figure 1**, **Figure 7**, **Figure 8**
**Method:** Seed Age: Treatment: Temperature: Seed source: Figures:	**C. Greenhouse LMA induction** Immature, tillers on intact plants Low humidity, cool vs warm treatment 18°C day/7.5 °C night vs 25°C day/18°C night Greenhouse-grown, on mother plant **Table 2**, **Figure 6**	

	PHS TRAIT	VIVIPARY TRAIT
**Method:** Seed Age: Treatment: Temperature: Seed source: Figures:	**D. Mature grain germination assay** Mature grain Seed germination, plating assay 22°C day/22°C night Greenhouse-grown grain **Figure 6**	**E. Immature grain germination assay** Immature grain Seed germination, plating assay 18°C day/7.5 °C night vs 25°C day/18°C night Greenhouse-grown grain **Figure 2**, **Figure 3**, **Figure 6**
**Method:** Seed Age: Treatment: Temperature: Seed source: Figures:	**F. Mature spike-wetting test** Mature, detached tillers Spikes under misting system 21-23°Cday/15-17°C night Field-grown detached spikes **Figure 5**	**G. Immature spike-wetting test** Immature tillers on intact plant Plants under misting system 18°C day/7.5°C night Greenhouse-grown spikes on intact plants **Figure 4**

**Figure 1:**
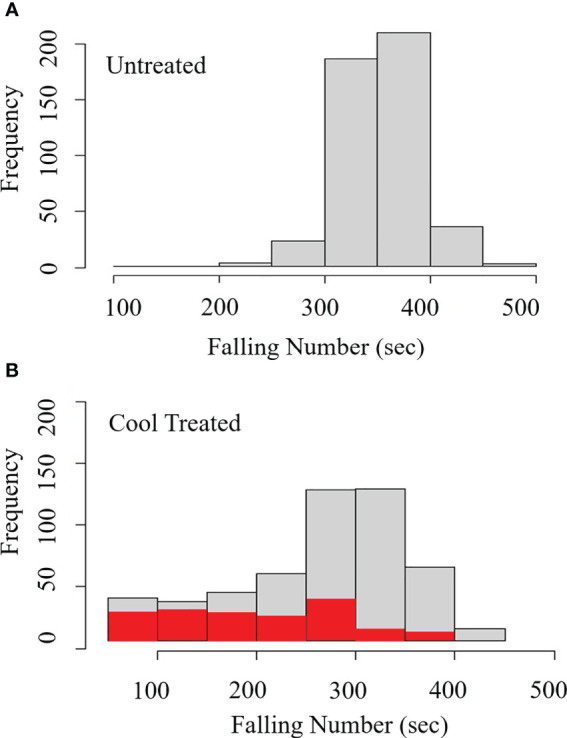
Visible germination was associated with falling numbers below 300 sec during cool induction of LMA without humidity control. Distributions show the frequency of lines at each grouping of FN (sec) (light grey) and frequency of lines showing visible germination (red) for a winter wheat population **(A)** without cool induction of LMA, and **(B)** with cool induction of LMA at an 18°C day/7.5°C night. During the cool induction, relative humidity was high reaching 50-70% during the day, and 80-95% during the night. The cool, high humidity treatment had a significant effect on the FN (p = 0.003), and FN below 300 sec was significantly associated with visible sprouting (p < 0.0001). n = 451 lines.

**Figure 2:**
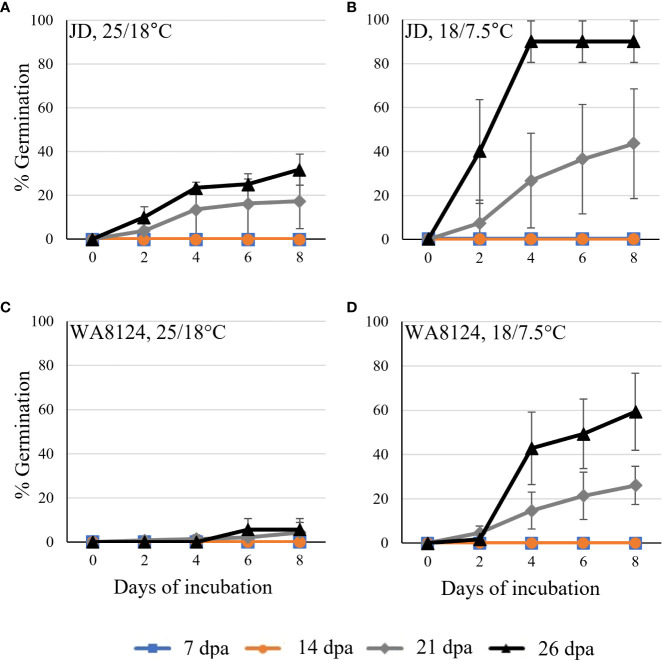
Developmental time course experiments showed wheat germination before physiological maturity. JD **(A, B)** and WA8124 **(C, D)** plants were grown under warm conditions, 25°C day/18°C night. Grain was collected at the indicated number of days past anthesis (dpa), plated on MS-agar, and incubated at the warm temperature 25°C day/18°C night **(A, C)** and cool temperature 18°C day/7.5°C night **(B, D)**. Three replications/spikes per treatment were plated with 30 grains from one spike per plate. Error bars represent standard deviation (SD).

**Figure 3:**
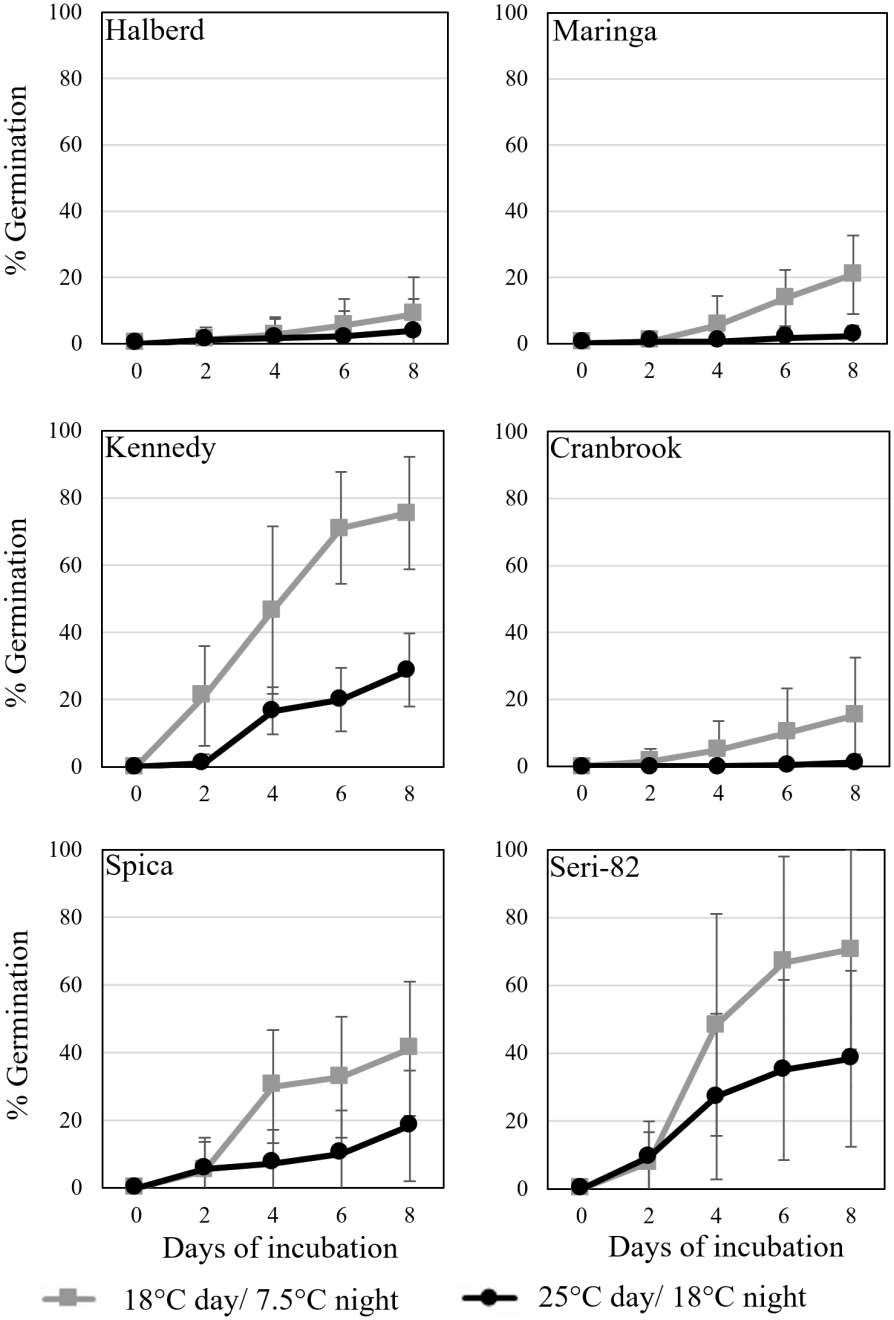
Vivipary occurred at a higher frequency at the cooler incubation temperature during plating experiments conducted at the soft dough stage of maturation. Grain was removed from spikes at 24-26 dpa, plated on MS-agar and incubated at either the warm (black) 25°C day/18°C night or cool (grey) 18°C day/7.5°C night treatment temperature. Mean percent germination of 12 replications of 30 grains per temperature per genotype is shown. Error bars represent SD.

**Figure 4:**
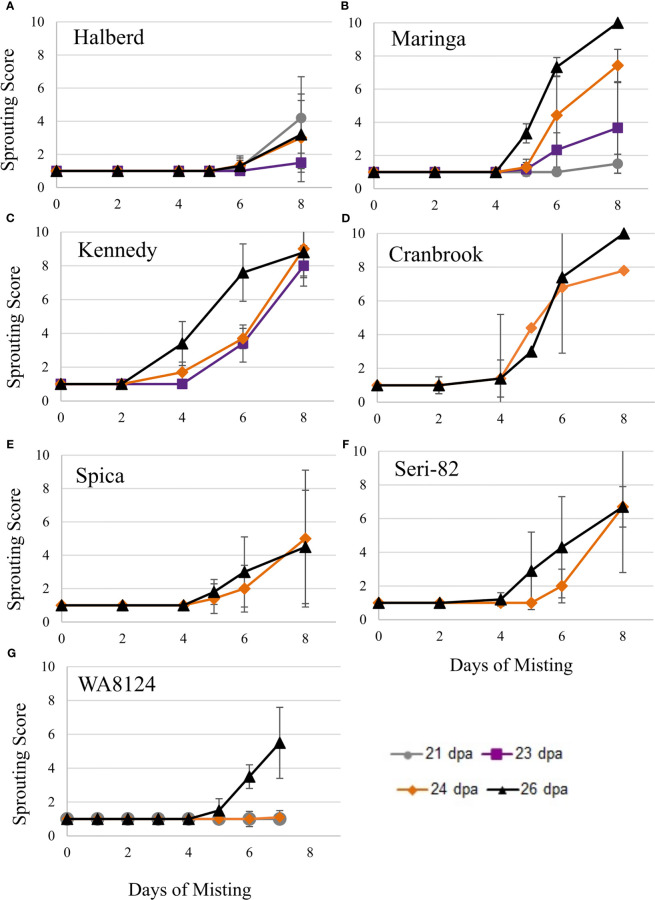
Immature spike-wetting tests. Spikes of wheat were tagged at anthesis in the greenhouse, then the mother plants carrying spikes at the soft dough stage of development (21 to 26 dpa) were placed under a misting system at 18°C day/7.5°C night (n = 3 to 9 spikes per developmental time point, from 3 to 5 mother plants). Not all time points were obtained for all varieties. Visible sprouting score was recorded every other day for **(A–F)** and daily for **(G)** WA8124. Error bars represent SD.

**Figure 5:**
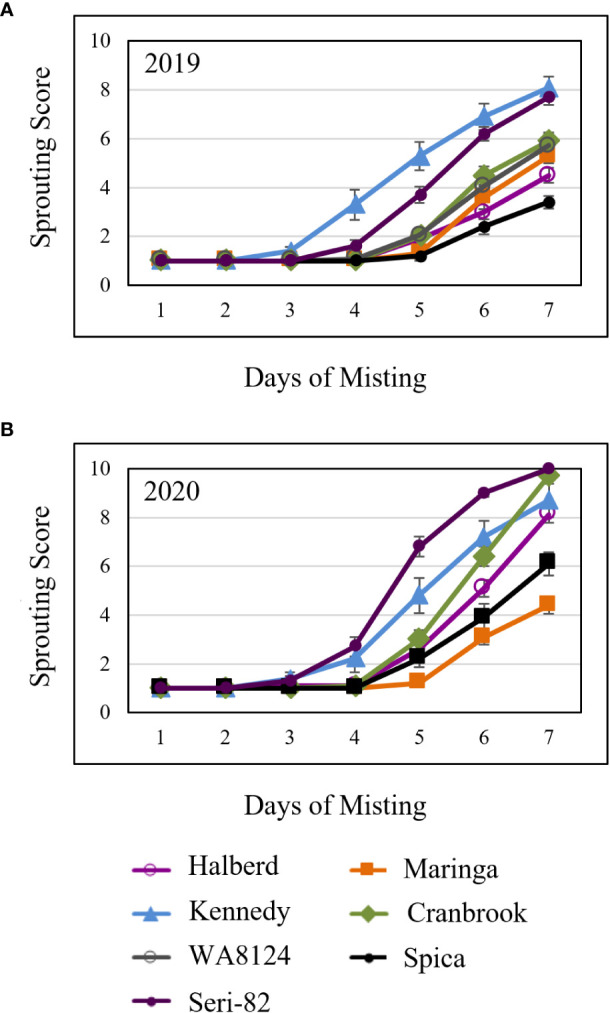
Mature spike-wetting tests. Spikes were harvested from the field at physiological maturity, allowed to after-ripen for 5 days, then placed under a greenhouse misting system in **(A)** 2019 and **(B)** 2020. Visible sprouting was scored daily. There were two field replications per year. n = 5 spikes per genotype per plot replication per year. Error bars represent standard error (SE).

**Figure 6:**
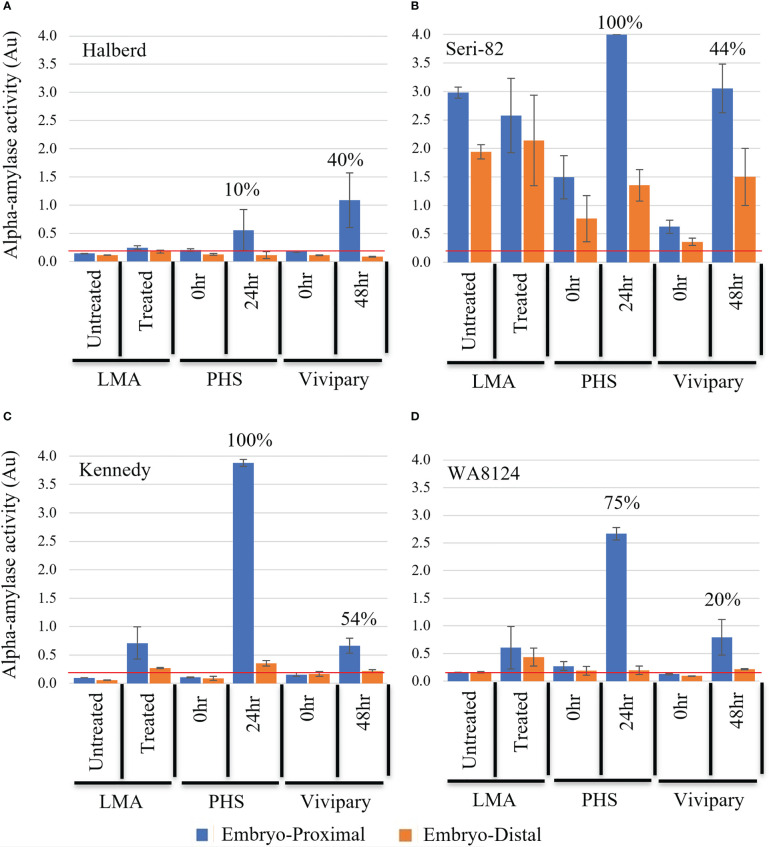
Distribution of α-amylase activity during LMA, PHS, and vivipary. Alpha-amylase activity was measured in absorbance A_620_ units (Au) at the embryo-proximal (blue) and embryo-distal (orange) halves of the grain following: 1) an LMA-induction experiment, 2) a mature grain germination experiment (PHS) incubated for 0 or 24 hr at 22°C, and 3) a vivipary plating assay conducted at 26 dpa at the cool temperature 18°C day/7.5°C night for 0 and 48 hr. The experiment was performed in LMA-resistant line **(A)** Halberd, LMA-constitutive line **(B)** Seri-82, and LMA-inducible lines **(C)** Kennedy and **(D)** WA8142. The mean percent germination is shown above the enzyme activity for the vivipary and PHS experiment. A value below the red line at 0.2 Au is estimated to result in an FN above 300 sec. Each bar represents 3 repetitions of 20 seeds. Error bars represent SE.

**Figure 7:**
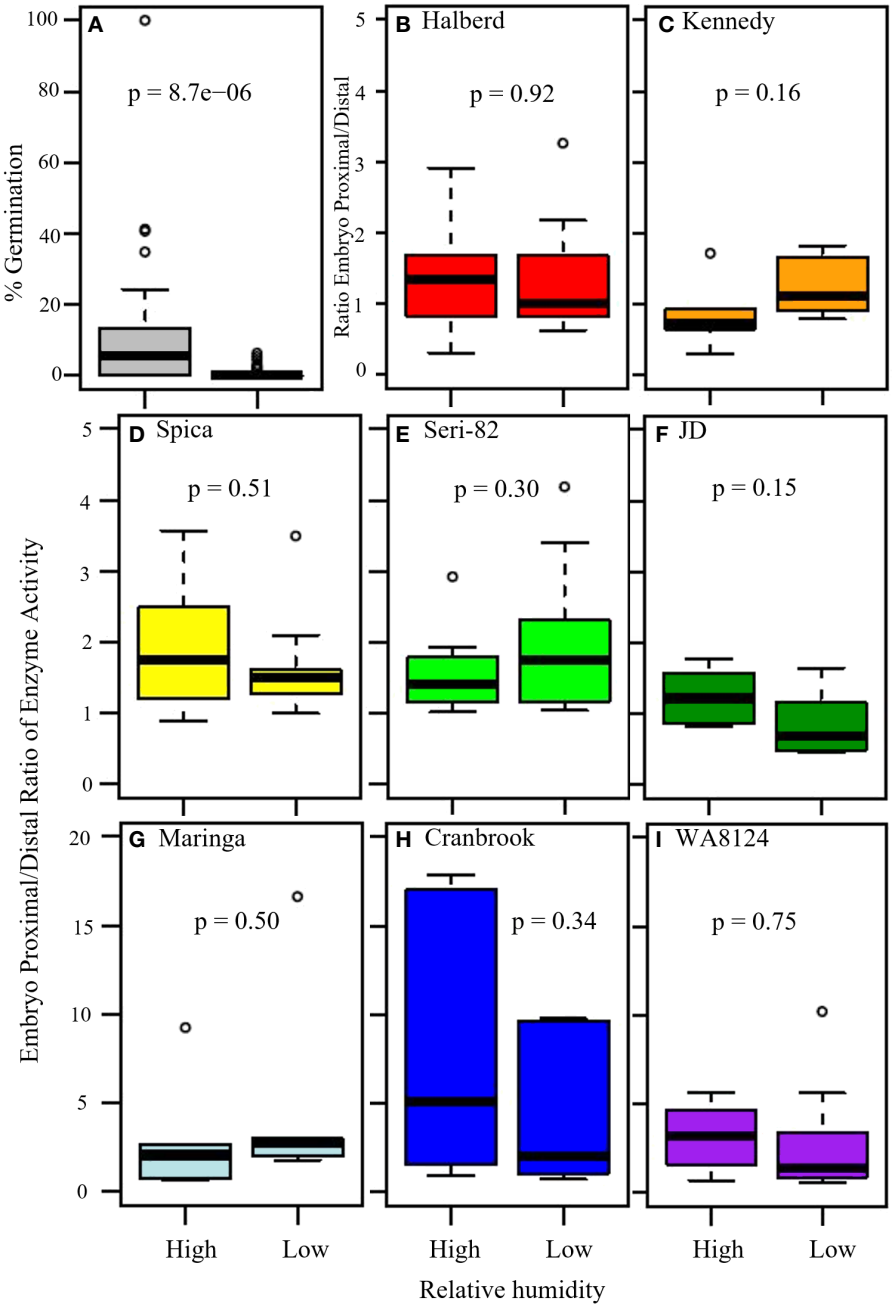
The effect of high versus low humidity on vivipary and the ratio of α-amylase enzyme activity at the embryo-proximal to embryo-distal half-kernels. **(A)** The effect of high versus low relative humidity on the percent germination of cool-treated detached wheat tillers (n = 15 lines). Tillers were placed in a cool chamber (18°C day/7.5°C night) at the soft dough stage of grain development for 7 days either with a dehumidifier (low humidity, 40-75%) or without (high humidity, 54-95%). A comparison of the ratio of α-amylase activity (Au) at the embryo-proximal to the embryo-distal end of the kernel showed similar ratios at vivipary-inducing high humidity and at the LMA-inducing low humidity treatment in indicated lines **(B–I)**. The average α-amylase enzyme activity for samples of 20 embryo-proximal and 20 embryo-distal half-kernels was determined for 6 tillers per treatment. Box and whisker plots (n = 6) are shown for the embryo-proximal activity divided by the embryo-distal activity. Displayed p-values were from Student’s t-test between the two treatments.

**Figure 8:**
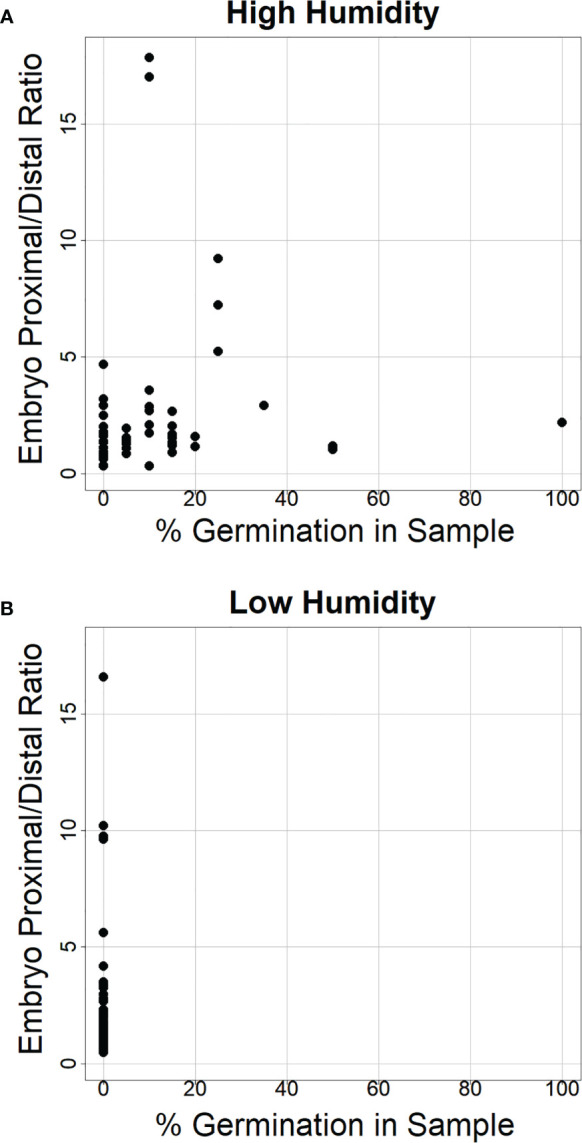
The ratio of α-amylase enzyme activity at the embryo-proximal to embryo-distal half-kernels does not appear to depend on the percent germination in the sample. Comparison of the ratio between the embryo-proximal and embryo-distal half-kernel α-amylase enzyme activities (Au) after cool treatment at high relative humidity **(A)** and low humidity **(B)** was plotted relative to the percentage of visible germination within the samples assayed for α-amylase enzyme activity. All spikes for all eight genotypes were plotted to examine whether higher germination was associated with a higher ratio.

### 2.2 Plant materials and growth conditions

A total of 451 lines from the 469 lines of the soft white winter quality association mapping (QAM) panel (Martinez et al., 2018) were used in an initial field LMA induction experiment. The panel was planted in 8 m^2^ plots at the Washington State University Spillman Agronomy Farm in Pullman, WA. A single replicate of each line plus 18 plots of ‘Madsen’ as a repeated control were planted as described in Martinez et al., 2018.

LMA, vivipary, and PHS traits were characterized in eight lines including six Australian cultivars, plus two Washington State wheat lines chosen to represent a range of LMA phenotypes based on previous work. Varieties obtained from the National Small Grains Collection included: LMA resistant ‘Halberd’ (PI377885; Mrva and Mares, 2001a) and ‘Maringa’ (PI584924; Barrero et al., 2013), and LMA susceptible ‘Kennedy’ (PI420949; Mrva et al., 2006; Liu et al., 2021b), ‘Seri-82’ (PI591774; Tan, 2004), ‘Cranbrook’ (PI495816; Mrva and Mares, 2001a), and ‘Spica’ (PI213830; Barrero et al., 2013). LMA susceptible soft white spring breeding line WA8124 (IDO599/S2K00095; Liu et al., 2021b) and LMA resistant spring club cultivar ‘JD’ (PI 656790; Liu et al., 2021b) were obtained directly from the Washington State University spring wheat breeding program. An additional seven spring lines were used for a high versus low humidity detached tiller vivipary/LMA-induction experiment including Washington State University breeding lines WA8148, HR07024-5, ‘Babe’, University of California UC1599, ‘AC-Cadillac’, ‘Waskada’, and University of Minnesota MN06075-4 (Liu et al., 2021b).

Field mature spike-wetting tests (**Table 1**) were performed for the eight lines grown at Spillman Farm in 1.5 m long paired head rows in 2019 and in 6 m plots in 2020. Field LMA induction experiments (**Table 1**) were performed for the eight lines shown in 6 m long plots in 2019 and 2022. The field planting in 2022 was used for the high versus low humidity experiment (**Table 1**) Two complete replicate blocks were planted on April 29, 2019, April 13, 2020, and April 29, 2022, and maintained as described in Schillinger et al., (2006).

All greenhouse, growth chamber, and germination experiments were conducted under a 16 hr. day/8 hr. night diurnal cycle. In greenhouse and growth chamber experiments using the eight lines, plants were grown in 3 L pots (one plant/pot) containing moistened Sunshine Mix-1 soil. Glasshouse-grown plants were grown under a light intensity of 300-400 μmol/m^2^/s maintained using supplemental sodium lamps. The glasshouse was maintained at a 21 to 24°C day and 15 to 18°C night temperature.

### 2.3 LMA induction

LMA was induced as described in Liu et al., (2021a): (1) under controlled environment conditions performed in a greenhouse using tillers on intact plants to confirm the originally published LMA phenotype in section 3.2 (**Tables 1**, **2**) and (2) under field conditions using detached tillers to evaluate the QAM panel and to obtain LMA- or vivipary-induced grain for half-kernel assays in sections 3.1 and 3.5 (**Table 1**).

**Table 2:** LMA Induction of α-amylase Activity.

Exp	Genotype	Untreated^f^	Treated^f^	T/U^a^	p-value^b^	N, n ^c^	N, n^d^	% G^e^
1	Halberd	0.11 ± 0.03	0.12 ± 0.05	1.1	0.227	14, 2	18, 5	0.0
1	Maringa	0.11 ± 0.03	0.14 ± 0.03	1.3	0.026	15, 4	15, 4	0.0
1	JD	0.14 ± 0.02	0.53 ± 1.07	3.8	0.181	4, 1	7, 2	0.0
1	Kennedy	0.15 ± 0.08	0.69 ± 0.57	4.6	6.0x10^-4^	10, 4	17, 4	0.0
1	Cranbrook	0.16 ± 0.06	2.65 ± 1.43	16.6	0.047	8, 3	3, 1	0.0
1	Seri-82	0.67 ± 0.53	2.81 ± 1.27	4.2	5.0x10^-5^	8, 2	12, 4	0.0
1	Spica	0.80 ± 0.48	–	–	–	30, 9	-, -	0.0
2	Halberd	0.12 ± 0.04	0.13 ± 0.04	1.1	0.208	15, 4	19, 6	0.0
2	Maringa	0.18 ± 0.04	0.26 ± 0.10	1.4	0.028	3, 1	14, 5	0.0
2	JD	0.19 ± 0.06	0.26 ± 0.13	1.4	0.131	3, 1	7, 2	0.0
2	Kennedy	0.11 ± 0.02	0.20 ± 0.17	1.8	0.383	7, 3	13, 5	0.0
2	Cranbrook	0.16 ± 0.10	0.23 ± 0.23	1.4	0.191	24, 7	24, 5	0.0
2	WA8124	0.17 ± 0.04	0.29 ± 0.19	1.7	0.008	3, 1	15, 4	0.0
2	Seri-82	0.80 ± 0.33	1.58 ± 1.07	2.0	0.021	7, 4	11, 4	0.0
2	Spica	1.81 ± 0.73	2.67± 0.84	1.5	0.009	15, 4	10, 2	0.0
3	Halberd	0.19 ± 0.05	0.22 ± 0.06	1.0	0.036	15, 4	16, 3	0.0
3	Maringa	0.26 ± 0.07	0.29 ± 0.06	0.9	0.012	35, 8	24, 4	0.0
3	Kennedy	0.21 ± 0.04	0.43 ± 0.29	2.0	5.9x10^-4^	13, 4	24, 5	0.0
3	Cranbrook	0.18 ± 0.22	1.32 ± 1.34	7.3	0.021	35, 10	12, 4	0.0
3	WA8124	0.18 ± 0.09	0.49 ± 0.37	2.0	0.002	9, 3	17, 3	0.0
3	Seri-82	2.56 ± 0.64	2.25 ± 0.68	0.8	0.096	18, 7	14, 3	0.0
3	Spica	1.94 ± 0.80	1.89 ± 0.97	1.1	0.433	39, 8	22, 3	0.0

a T/U is the ratio of the treated divided by the untreated α-amylase activity measured in Au.

b p-value is showing if there is a significant difference between treated and untreated based on a Student’s *t*-test.

c N is the number of spikes untreated. n is the number of plants untreated.

d N is the number of spikes treated. n is the numbers of plants untreated.

e %G is the percent grain germination observed before running α-amylase enzyme assays.

f Average enzyme activity (Au) + (plus-minus) standard deviation.

(1) *Controlled environment LMA induction* (**Table 1**). Briefly, plants were grown in the glasshouse until heading then were transferred to the basal temperature of 25°C day and 18°C night in a Conviron™ growth room (GR96) using sodium lamps at 300 μmol/m^2^/s to maintain a 16 hr. day/8 hr. night. Spikes were tagged at anthesis, the point at which the anthers shed their pollen. Only the first three main tillers were used for experiments. The number of replications per treatment is given in **Table 2**. Intact plants were shifted to a Conviron™ growth chamber (PGR15 or GR96) using the same diurnal lighting conditions at the cool treatment temperature of 18°C day and 7.5°C night when spikes ranged from 22-to-26 days post anthesis (dpa) which is equivalent to 451 to 602 growing degree days past anthesis (GDDpa) at this temperature. The chamber relative humidity was maintained between 40% and 75% using an EVA-Dry™ dehumidifier based on a HOBO datalogger readings (day 38-55%, night 62-75%). After a seven-day cool treatment, the plants were returned to the basal growth temperature. The untreated plants remained at the basal growth temperature for their entire experiment. Once the plants fully matured, individual spikes were threshed and 20 grains from each spike were milled in a coffee grinder and Phadebas α-amylase enzyme assays were performed (as in Hauvermale et al., 2023). Temperature and relative humidity (RH) were recorded using an Onset™ HOBO™ temperature and RH logger (UX100-003).

(2) *Field LMA induction* (**Table 1**). Rather than tagging single spikes at anthesis, the date on which 50% of a plot reached anthesis was recorded. At the soft dough stage at approximately 500 GDDpa (Miller et al., 2001; Liu et al., 2021a), 15-20 spikes per plot were cut, tied, then placed immediately into a bucket of water (**Supplementary Figure 1**). Bundles of tillers were incubated for 7 days with an 18°C day/7.5°C night in Reliance LED™ incubators (Port Townsend, WA; https://www.reliancelabs.com/). Afterwards, bundles were moved outside to senesce under warm conditions. Untreated controls were left outside at ambient temperature in buckets of water until they senesced. In 2017, a single field replicate of the QAM panel was sampled for each treatment. Bulked samples of 15 to 20 spikes were threshed, milled in a UDY mill and analyzed using either the falling numbers method (as in Martinez et al., 2018) or the Phadebas enzyme assay (as in Hauvermale et al., 2023). After 2017, bucket lids were modified to prevent spike bundles from lying on top of each other, thereby reducing moisture with improved air circulation (**Supplementary Figure 1**). For low humidity LMA inductions (**Table 1**), an EVA-Dry dehumidifier was used to maintain RH <75% after 2017. A low humidity cool temperature treatment of detached tillers was performed using a dehumidifier to maintain the relative humidity between 40 and 75%. A high humidity cool temperature treatment of detached tillers was conducted without a humidifier. This resulted in vivipary induction (**Table 1**), and a relative humidity between 54 and 95%. In high humidity experiments, it was possible to see condensation form on the buckets overnight.

### 2.4 Germination capacity during a time course of grain development

To examine the moisture content during grain development in growth-chamber grown plants, a developmental time course experiment was performed on WA8124, JD, Halberd, and Seri-82. Kernels were collected at 7 dpa, 14 dpa, 21 dpa, 24 dpa, 26 dpa, 35 dpa, and 45 dpa when plants were grown under a 25°C day and 18°C night in a Conviron™ growth room (GR96) using sodium lamps at 300 μmol/m^2^/s to maintain a 16 hr. day/8 hr. night. Based on visual inspection, the kernels reached the milk stage at approximately 7-14 dpa, the soft dough stage at 21-26 dpa, hard dough stage at 28-30 dpa, physiological maturity at 35-40 dpa, and harvest maturity at 42-47 dpa (Heyne, 1987). Grains were dissected from the immature spike at each time point, placed in a sealed tube, and then grain moisture content was measured using a Perten NIR Model 7250 using the wheat calibration in the Results Plus software.

A developmental time course experiment was performed at 7 dpa, 14 dpa, 21 dpa, and 26 dpa to determine when wheat grain acquires the ability to germinate. Spikes were tagged at anthesis, then grains were dissected from the spike at the indicated developmental time point to avoid damaging the seed coat. Grains were kept in a tube containing a moist Kimwipe to maintain moisture until plating. The grains were sterilized using a 10% bleach (0.6% sodium hypochlorite) and 0.01% sodium dodecyl sulfate solution for 10 minutes with occasional shaking. Seeds were then washed 6 times with 15 mL sterile water. Using ethanol-cleaned tweezers, 30 seeds per spike were evenly placed on an MS-agar plate (0.5x Murashige and Skoog basal medium (Sigma-Aldrich) with 0.8% agar (Invitrogen)) in order to provide nutrients for immature germinating grains. Plates were sealed with Parafilm. For each genotype, 3 replicates of 30 grains each (taken from 3 independent spikes) were plated at the warm (25°C day and 18°C night) and at the cool temperature (18°C day and 7.5°C) for each developmental time point. Germination was scored at 2, 4, 6, and 8 days of incubation. Two Percival™ (IntellusUltra C8, model CU22L) incubators were set to the warm and cool incubation temperature with a 16 hr. day and 8 hr. night using fluorescent lamps at 150 μmol/m^2^/s.

### 2.5 Vivipary germination assays

Vivipary germination assays (**Table 1**) were used to examine germination capacity of threshed kernels prior to maturity as described for the time course experiment above but restricted to time points during the soft dough stage (Zadoks stage 83-85) of grain maturation from 24 to 26 dpa (**Table 1**). Germination was scored over 7 days of incubation at the cool (18°C day and 7.5°C) or warm (25°C day and 18°C night) temperature maintained in Percival incubators (model CU22L and E30B). The higher temperature of 25°C day/18°C night was selected to match the basal conditions that give little LMA induction, whereas the low temperature of 18°C day/7.5°C night was previously shown to efficiently induce LMA (Liu et al., 2021a). Two spikes (one to be incubated at the warm, and one at the cool temperature) from each of three separate plants were harvested per genotype at the soft dough stage, hand threshed, sterilized, and plated on MS-agar as described in section 2.4. The vivipary experiment was repeated twice with small differences in the culture of the mother plant. In the first experiment, the mother plants were grown under glasshouse conditions with no vernalization. To increase the synchrony of flowering of the mother plants in the second supplementary experiment, seeds were cold stratified at 4°C for 24 hr. on 5 mM MES pH 5.5 moistened-filter paper in a petri dish before germination at room temperature for 48 hr., then vernalized at 4°C for 7 days. In the second experiment an additional three spikes derived from 3 different plants were plated for collection at 0 hr. and 48 hr. of incubation for use in half-kernel α-amylase enzyme assays.

### 2.6 Spike-wetting tests to detect preharvest germination of mature and immature grain

To examine preharvest sprouting tolerance, spike-wetting tests were performed as described in Martinez et al., 2018 on mature spikes of the spring wheat lines Halberd, Maringa, Kennedy, Cranbrook, Spica, Seri-82 and WA8124 (**Table 1**). Briefly, spikes were harvested from the field at physiological maturity (Zadoks stage 91), allowed to after-ripen for 5 days, then stored at -20°C until placed under a misting system. Spikes were misted for 6 sec every minute and scored on days 3 through 7 using a modified scale based on McMaster and Derera, 1976 (**Supplementary Table 1**). No sprouting was observed on day 1 and 2 of misting.

To examine whether cool, moist conditions could induce vivipary, premature germination on the mother plant, spike-wetting tests were performed using intact plants during the soft dough stage of grain maturation (**Table 1**). Spikes were tagged at anthesis, while plants were growing under warm conditions (25°C day and 18°C night), then the plants were moved to a cool misting chamber when spikes were between 21 and 28-dpa. Plants were misted for 6 sec every minute in a Conviron™ growth chamber (PGR15) at a temperature of 18°C day and 7.5°C night under a 16 hr. day diurnal cycle, under sodium lamp light to 300 μmol/m^2^/s. The experiment was conducted twice. In the first experiment sprouting was scored on days 2, 4, 5, 6, and 8 of misting, whereas in the second experiment sprouting was scored daily through day 7 of misting. Sprouting was scored using the 1-10 scale described above (**Supplementary Table 1**; McMaster and Derera, 1976; Martinez et al., 2018). A sprouting score of 5 corresponds to a fully germinated spike and all scores higher than 5 refer to degree of post-germinative seedling growth.

### 2.7 Phadebas α-amylase enzyme assays

Alpha-amylase enzyme activity was measured in intact kernels using the 96-well Phadebas assay described by Hauvermale et al., 2023. To reduce variation between plates and batches of Phadebas™ reagent, every 96-well plate contained a standard curve of seven samples of known activity (0.12, 0.29, 0.50, 1.32, 1.68, 2.11, and 2.25 Au). Linear regression was used to normalize enzyme activity in Absorbance units (Au, Absorbance at 620 nm) to the known activity of the standards.

### 2.8 Half-kernel assays after induction of LMA, vivipary, and PHS

Half-kernel Phadebas assays were used to examine if viviparous kernels better resemble LMA or mature-germinating PHS kernels. Previous research showed that PHS results in ~10-fold higher α-amylase at the embryo-proximal than at the embryo-distal end of the kernel, whereas LMA typically results in α-amylase levels randomly distributed throughout the aleurone layer (Mrva and Mares, 1996; Mrva et al., 2006). The pattern of α-amylase activity was measured in half-kernels after induction of vivipary (**Table 1**), LMA (**Table 1**), and PHS (**Table 1**) based on the half-kernel method of Mrva and Mares, 1996.

Vivipary was induced using the plating method (**Table 1**). In Halberd, Seri-82, and Kennedy, three separate plates of 30 kernels each derived from three different plants were collected at 0 hr. and 48 hr. each. There were 3 technical replicates from each plate consisting of 10 kernels each. For vivipary induction of WA8124 only 3 replicates of 10 kernels each were generated. Mature grain germination (called “PHS” in section 3.5) was induced in three plates of 30 kernels each obtained by bulking grains harvested from six to eight parent plants. Both mature and immature kernels were sterilized and plated as described for vivipary germination assays (**Table 1**). Mature kernels were collected at 0 hr. and 24 hr. of imbibition. For LMA experiments, fully mature kernels from field LMA-inductions (**Table 1**) were subjected to half-kernel Phadebas assays. There were three replicate samples from LMA-induced and two replicate samples from the uninduced controls. Samples were bulked kernels from a single field plot. Kernels from each genotype, treatment and time point were freeze-dried for 24 hr. in a Labconco™ lypholizer to halt biological activity before performing enzyme assays.

To perform half-kernel Phadebas α-amylase enzyme assays, the ten kernels from each replicate sample were bisected transversely with a razor blade. The embryo-proximal half-kernels were placed in one tube and the embryo-distal half-kernels in another 1.5 mL tube. Due to the small sample size, kernels were ground in a mortar and pestle with liquid nitrogen. In cases where some samples were too small to obtain 0.2 g of meal, 0.1 g of meal was used in the assay and the activity measured doubled. The 96-well Phadebas assay was performed as described above.

### 2.9 Half-kernel assays after field induction of vivipary at high humidity and of LMA at low humidity

To examine α-amylase levels in a larger number of half-kernels exposed to LMA- or vivipary inducing conditions, a field detached tiller LMA-induction experiment (as in section 2.3) was performed using a high humidity cool treatment (18°C day/7.5°C night) to induce vivipary (**Table 1**) and a low humidity cool treatment to induce LMA (**Table 1**). Two cool 18°C day/7.5°C night chambers were filled with buckets of water regardless of the number of spikes present in order to raise the relative humidity in the chamber, one chamber with and one without a dehumidifier (Percival E3VL). Detached tillers were collected from the field at the soft dough stage of development (485-545 GDDpa), bundled, and then placed into buckets of water as shown in **Supplementary Figure 1B**. The cool treatment at each humidity level was performed for 3 tillers from each of one to three plots for each genotype (total of 3, 6, or 9 tillers per genotype). The low humidity LMA induction was performed using an incubator containing an Eva-Dry dehumidifier to maintain relative humidity levels 40-75%. The high humidity vivipary induction was performed using the same model of incubator, but the humidity range was 54-95%. Percent visible germination was counted for kernels from 3 to 9 spikes each from the high and low humidity treatments. Half-kernel enzyme assays were performed for the 8 genotypes of known LMA phenotype after the high and low humidity experiment. These lines had two or three plots each. Three spikes were sampled from each plot, so that 6-9 spikes were assayed for half-kernel α-amylase activity per genotype. For each spike, 20 kernels were bisected, and the embryo-proximal and -distal halves were placed in separate tubes. The number of germinated kernels sampled was recorded. Half-kernel assays were also performed for the mature grain “PHS” germination assays performed as described in section 2.8. Half-kernel assays were performed as described in section 2.7.

### 2.10 Statistical analysis

Linear models for analysis of variance (ANOVA) were performed using aov and Student’s *t*-test using t.test in the stats package of R (v 4.0.2) in RStudio (RStudio Team, 2020).

## 3 Results

### 3.1 Field LMA induction caused premature germination of wheat grain

Field LMA induction experiments were conducted on detached tillers from 451 lines of the QAM winter soft white wheat association panel in 2017. Spikes cut from the field at the soft dough stage of grain development were subjected to a 7-day cool treatment (18°C day/7.5°C night) to induce LMA (**Supplementary Figure 1A**). The LMA cool treatment resulted in a significant reduction in FN indicating elevated α-amylase (p = 0.003; **Supplementary Table 2A**); such that 59% (n = 451) of the cool-treated samples showed an FN below 300 sec whereas 5.3% (n = 451) of the untreated samples showed an FN below 300 sec (**Figures 1A, B**). In the process of preparing grain for falling number testing, it was observed that many of the cool-treated samples had >10% visibly sprouted grains, suggesting that low FN resulted from germination rather than from LMA induction. This was surprising because spikes were collected prior to physiological maturity and because the growth chambers did not include a misting system. Whereas daytime humidity was 50-70%, nighttime relative humidity rose to 80-95% in LMA-induction chambers with an 18°C day and 7.5°C night. The elevated nighttime humidity likely led to moisture condensation on spikes and stimulated grain germination before physiological maturity, a phenomenon called vivipary (McCarty, 1995). The cool treatment was significantly associated with precocious germination when samples had an FN below 300 sec (p = 2.0 x 10^-16^; **Supplementary Table 2B**), suggesting that germination resulted in α-amylase expression. Only 7 untreated samples showed visible sprouting, whereas 134 of the cool-treated samples showed visible sprouting (red bars in **Figures 1A, B**). Among cool-treated samples, 44% of samples with an FN <300 sec were visibly sprouted whereas 9.6% of the samples with FN >300 sec were visibly sprouted. As expected, low FN was significantly associated with elevated α-amylase activity when measured in the 265 lines with an FN below 300 sec using Phadebas™ enzyme assays (r = -0.72, p <2.0 x 10^-16^) (Liu et al., 2021b). These results suggest that low FN and elevated α-amylase can result from premature germination rather than LMA if the humidity rises during the cool-treatment used to induce LMA. To clearly differentiate between LMA and vivipary, subsequent LMA induction experiments were conducted using a dehumidifier to maintain a chamber relative humidity below 75% (Liu et al., 2021a; Liu et al., 2021b; this study) and using buckets with modified lids to keep wheat bouquets separated and facilitate air circulation (**Supplementary Figure 1B**).

### 3.2 The LMA phenotype of eight wheat cultivars

To examine whether LMA could be induced without also triggering vivipary, LMA was induced in spikes still attached to the mother plant using growth chambers containing a dehumidifier to maintain humidity below 75% (as in Liu et al., 2021a; Liu et al., 2021b; **Table 1**). Three consecutive LMA experiments were conducted (**Table 2**). One plant showing the expected LMA phenotype in each experiment was advanced to obtain the plants used in the following experiment in order to obtain progeny with confirmed LMA phenotype for use in subsequent vivipary experiments. Based on an ANOVA, genotype and dpa were significant sources of variation in α-amylase activity (**Supplementary Table 3**). Based on failure to induce LMA, Halberd showed the strongest LMA resistance whereas Maringa and JD showed moderate resistance. Previous work showed that Spica has a constitutive LMA phenotype resulting in significant α-amylase activity both with and without cool-treatment (Barrero et al., 2013). In addition to Spica, an LMA constitutive phenotype was also observed in the cultivar Seri-82 (25°C day/18°C night), which was previously shown to be LMA susceptible but not constitutive (Tan, 2004). While cool treatment was not required to observe α-amylase expression in Spica and Seri-82, it enhanced the expression of α-amylase by 1.5- or 2-fold in experiment 2. Within an ANOVA of all 8 genotypes (**Supplementary Table 3**), the factors experiment and plant did not have a significant effect on LMA induction. However, there was variation in α-amylase induction in varieties requiring cool-temperature induction of LMA, Cranbrook and Kennedy (**Table 2**). The high degree of variation between experiments and plants of the same genotype is consistent with previously published work (Mares et al., 1994; Mrva and Mares, 2001b; Liu et al., 2021a, b; reviewed in Mares and Mrva, 2008). When cool-treated samples were examined, no apparent visible sprouting was observed (**Table 2**, n > 75 grains per cultivar from 3 treated spikes). Thus, when relative humidity is maintained below 75%, α-amylase activity can be induced by cool treatment during grain maturation without also inducing visible sprouting in cultivars that induce vivipary under cool, humid conditions.

### 3.3 Vivipary during the grain maturation stage of wheat development

Vivipary germination assays were performed on MS-agar plates to examine the phenotype without the covering tissues of the spike. Germination assays were performed at four time points during grain development to examine when wheat becomes able to induce vivipary (**Table 1**). Two cultivars were examined, JD and WA8124. Grain was plated on MS-agar to provide nutrients for germinating immature grains and to examine susceptibility to vivipary in terms of percent germination. Germination assays were performed at a higher (25°C day/18°C night) and lower (18°C day/7.5°C night) temperature to determine if the low temperature typical of LMA induction enhanced premature germination (**Figure 2**). No germination was observed during the milk stage of grain development at 7 and 14 dpa, whereas grain at the soft dough stage of 21 or 26 dpa showed germination. The cooler incubation temperature was associated with more efficient germination, consistent with the observation that grain was more prone to vivipary during cool-temperature induction of LMA than at a higher temperature (**Figure 1**). Similar results were obtained in an independent experiment using Seri-82 (**Supplementary Figure 2**). Based on this result, subsequent vivipary experiments focused on the soft dough stage of grain development (Zadoks stage 85).

In a parallel experiment, percent grain moisture was determined at 7, 14, 21, 24, 26, 35, and 42 dpa in WA8124, JD, Halberd, and Seri-82 as an objective measure of progress in grain development (**Supplementary Figure 3**). Grain at 7 dpa ranged from 45-65% moisture content, at 14 dpa ranged from 42-55% moisture content, whereas grain between 21 and 26 had 32-46% moisture content. Grain at physiological maturity, 35 to 40 dpa tended to have 23-36% moisture content, and mature grain at 42 dpa had about 11% moisture content.

Vivipary was characterized in the remaining six lines characterized for LMA in **Table 2** using spikes collected at 24-26 dpa. Spikes were collected from plants growing at 25°C day/18°C night and grains were hand-dissected from the spikes. Grain was germinated under warm (25°C day/18°C night) or cool (18°C day/7.5°C night) conditions (**Figure 3**; **Table 1**). The cool incubation temperature is the same used to induce LMA. The experiment was repeated to examine the consistency of the phenotype (**Supplementary Figure 4**). In all eight cultivars, cool temperatures significantly stimulated premature germination compared to warm temperatures at 24-26 dpa (p-value = 0.006, **Supplementary Figure 4**). Halberd, Maringa, Spica, and Cranbrook all showed some degree of resistance to germination both at cool and warm temperatures. Seri-82 and Kennedy showed greater susceptibility to vivipary both at the warm and cool incubation temperature.

### 3.4 Characterization of premature grain germination/vivipary and of mature grain preharvest sprouting in spike-wetting assays

To confirm that wheat grain can undergo vivipary while still on the mother plant in response to misting at the cool temperature (18°C day/7.5°C night) used to induce LMA, spike-wetting assays were performed using spikes still attached to the mother plant when spikes were at the soft dough stage of grain maturation at 21 to 26 dpa (**Table 1**). A misting system was used to obtain more uniform moisture conditions than could be obtained with the detached-tiller LMA induction procedure used in **Figure 1**. The experiment was performed using a panel of cultivars with published LMA phenotypes: Cranbrook, Kennedy, Spica, WA8124, and Seri-82 are LMA susceptible, whereas Maringa and Halberd are resistant (Mrva and Mares, 2001a; Mrva et al., 2006; Barrero et al., 2013; Liu et al., 2021a). Plants were placed under misters at 18°C day/7.5°C night for 8 days and visible sprouting scored on days 2, 4, 5, 6, and 8 using a 1-10 sprouting scale where 1 means no sprouting and a 10 shows uniform coleoptile emergence associated with emergence of the first leaf (**Supplementary Table 1**; McMaster and Derera, 1976; Martinez et al., 2018). Although all cultivars were able to induce vivipary to some degree at the soft dough stage, Halberd, WA8124, and Spica appeared more resistant (**Figure 4**). Sprouting score appeared to generally increase later in seed development (with increasing dpa), suggesting that germination capacity increased during maturation between 21 and 26 dpa (**Figure 4**). Of the LMA-resistant lines, Halberd was strongly resistant, whereas Maringa was moderately susceptible (**Figures 4A, B**). Of the LMA-susceptible lines, Kennedy and Cranbrook were strongly susceptible to vivipary, whereas Spica and Seri-82 were only moderately susceptible to vivipary (**Figures 4C–F**). Thus, vivipary results did not always correspond to LMA phenotype.

To compare susceptibility to vivipary with mature grain preharvest sprouting, spike-wetting tests were also performed on mature spikes harvested from the field at physiological maturity and after-ripened for 5 days in 2019 and in 2020. Mature spike-wetting tests were performed under normal greenhouse temperatures (21-24°C day and 15-18°C night) instead of the cool temperatures used to induce vivipary with misting (**Figures 5A, B**). Mature spikes showed similar sprouting susceptibility to premature spikes (**Figures 4**, **5**). For example, Halberd was resistant to both vivipary and PHS, whereas Kennedy and Seri-82 were susceptible to both vivipary and PHS. Maringa, however, appeared to be more resistant to PHS than to vivipary.

### 3.5 The pattern of α-amylase expression during vivipary

Alpha-amylase activity was measured in half-kernels of Halberd, Seri-82, Kennedy, and WA8124 to examine whether the pattern of expression during vivipary resembled LMA or mature grain germination. During mature grain germination, α-amylase levels are higher at the embryo-proximal than -distal end of the grain, whereas during LMA α-amylase levels are more evenly distributed (Mrva and Mares, 1996). Immature grains were incubated at 18°C day/7.5°C night during the experiment shown in (**Supplementary Figure 4**), except those grains were lyophilized after 0, 24, and 48 hours of imbibition (**Table 1**). Grains were then cut in half and the α-amylase enzyme activity measured in embryo-proximal and embryo-distal ends of the grain (**Figure 6**). Only the 0 hr. and 48 hr. time points are shown because no increase in α-amylase activity was observed at 24 hr. As a control, half-kernel assays were also performed on grain from the 0 hr. and 24 hr. time points of a mature-grain germination experiment (**Table 1**) referred to as “PHS” for simplicity in (**Figure 6**) and from an LMA induction experiment (**Table 1**) where “LMA”-induced grains were cool-treated and “untreated” were not.

Halberd showed no α-amylase expression with LMA-induction but did show variable α-amylase expression at the embryo-proximal end of the grain at the vivipary 48 hr. and PHS 24 hr. time points (**Figure 6A**). Thus, α-amylase expression during vivipary better resembled mature grain germination than LMA in Halberd.

Seri-82 expresses LMA constitutively but at varying levels (**Table 2**). This is likely why the starting α-amylase levels in the 0 hr. timepoint of the vivipary experiment were lower than in the untreated control from the LMA experiment (**Figure 6B**). In the vivipary experiment, there was greater than a 3-fold increase in α-amylase levels at the embryo-proximal ends of the grain at 48 hr., whereas the embryo-distal α-amylase level was highly variable. A stronger and more consistent increase in α-amylase was observed in the embryo-proximal end at 24hr of the mature grain PHS experiment. Because of the variability in α-amylase expression, it is not possible to conclude whether vivipary in Seri-82 better resembles LMA or mature grain PHS.

Kennedy and WA8124 can induce LMA in response to cool-treatment (**Table 2**; Liu et al., 2021a). Consistent with this, there was no α-amylase expression in either the untreated control from the LMA experiment nor from the 0 hr. time points of the vivipary and PHS experiments (**Figures 6C, D**). In Kennedy at the 48 hr. vivipary time point, α-amylase expression was induced at the embryo-proximal but not the embryo-distal end of the grain (**Figure 6C**). However, the level of α-amylase expression was lower during vivipary than during mature grain germination at the “24 hr. PHS” time point. In WA8124, α-amylase expression was also more strongly induced at the embryo-proximal than embryo-distal end of the kernel during vivipary (**Figure 6D**). However, in the mature grain “PHS” experiment, there is a much higher α-amylase expression level at the embryo-proximal end than in the vivipary 48hr time point. These results show that α-amylase is expressed during vivipary, indicating that vivipary may contribute to low FN in the field. In **Figure 6**, it is difficult to judge if embryo-proximal α-amylase levels are lower during vivipary than PHS because vivipary is more like LMA or because germination rates are lower during vivipary based on a limited number of lines. To address this, α-amylase levels were examined in a larger population after a field LMA/vivipary induction experiment.

To examine whether high humidity induced vivipary at the soft dough stage at significantly higher levels than low humidity, a field detached-tiller 7-day cool treatment was performed in incubators at high (**Table 1**, 54-95%) and low relative humidity (**Table 1**, 40-75%). This experiment was performed using the 8 genotypes with known LMA phenotypes plus an additional seven spring wheat lines (see Methods section 2.2). For each genotype, 3 to 9 individual spikes were hand-threshed and percent germination was calculated on a per spike basis (**Supplementary Table 4**). The high humidity condition resulted in a significantly higher percentage of visibly sprouted kernels (vivipary) across all 15 genotypes (**Figure 7A**; n= 102 spikes per treatment, p-value = 5.0 x 10^-9^). This experiment demonstrated that the phenomenon of vivipary observed in **Figure 1** could be reproduced in another field season and in different genotypes. It should be noted that the low humidity condition greatly reduced but did not eliminate germination under the detached tiller incubation conditions. Rare germination events were observed, even at low humidity (**Supplementary Table 4**).

To examine whether the percent germination at high humidity was correlated to the germination at low humidity, the percent germination of all field replications (averaged by plot) for 15 genotypes were compared at high and low humidity (**Supplementary Table 4**). There was a high degree of variation in germination at high humidity, and higher germination at high humidity did not appear to be associated with the rare germination observed at low humidity (**Supplementary Figure 5**, n=40). This suggests that the rare instances of germination at low humidity are not necessarily indicative of a tendency to vivipary at high humidity. Thus, we recommend that either plating experiments as in **Figure 3** or high-humidity cool-treatment be used to characterize vivipary.

The field cool-induction of vivipary and LMA enabled the comparison of embryo-proximal to -distal α-amylase ratios in a larger number of lines without plating on MS-agar to induce vivipary. The expectation being that if vivipary is more like PHS than LMA, then there should be higher levels of α-amylase close to the embryo resulting in a higher embryo-proximal to embryo-distal ratio. Although the ratio sometimes ranged higher in the high humidity/viviparous sample than in the low humidity/less germinated sample (for example, Spica, JD, Cranbrook, and WA8124), there was not a statistically significant difference between the high and low humidity ratios in any of the eight genotypes (**Figures 7B–I**). Three lines, WA8124, Cranbrook, and Maringa had higher ratios than the other lines, however, there was not a statistically significant difference between the ratios at low and high humidity suggesting that this difference was a property of the genotype rather than a result of the treatment (p > 0.05; **Figures 7G–I**). The difference between vivipary and PHS/mature grain germination was particularly evident when half-kernel assays were also performed on the 24 hr. PHS time point of each line (**Supplementary Figure 6**). Ratios were much higher in germinating mature grains. This suggests that the regulation of α-amylase during vivipary is more similar to LMA than to PHS.

Consistent with the idea that visible premature germination/vivipary did not result in a substantially higher embryo-proximal than -distal α-amylase level, there was no clear correlation between the percent germination in the sample assayed for enzyme activity and the embryo-proximal/distal ratio (**Figures 8A, B**). While there was a great deal of spike-to-spike variation in ratio, the range in ratios was similar in the low and high humidity experiments. Taken together, these data suggest that vivipary, seed germination before the seed has become quiescent, results in α-amylase expression patterns similar to LMA.

## 4 Discussion

This study discovered that precocious germination, termed vivipary can occur in wheat during the maturation phase of grain development. Wheat vivipary can occur both in plating assays and on the mother plant under high humidity, suggesting that vivipary is another potential cause of low falling numbers in wheat fields. The fact that vivipary occurs at cooler temperatures indicates that it is important to control humidity during LMA-induction experiments to differentiate between the two problems. Time course experiments showed that cooler temperature stimulated vivipary during the soft to hard dough stage of grain maturation (**Figures 2**–**4**; **Supplementary Figures 2–4**). This observation was consistent with a previous study showing that one wheat genotype could germinate when plated during grain maturation, especially at lower temperatures (Ichinose et al., 2002). Subsequent experiments examined cultivars known to vary for LMA phenotype, so that we could compare susceptibility to LMA and vivipary.

There was some degree of correspondence between LMA, PHS, and vivipary phenotypes. For example, Halberd had resistance to all three whereas Seri-82 was susceptible to all three phenomena. However, there were clear counterexamples suggesting that they can be distinct phenomena. Maringa showed moderate resistance to both LMA and PHS but was susceptible to vivipary while on the mother plant (**Figures 4**, **5**; **Table 2**; **Supplementary Table 4**). Spica appeared to be resistant to both PHS and vivipary in spike-wetting tests but was highly LMA susceptible and moderately susceptible to vivipary in plating assays (**Figures 3**–**5**; **Table 2**; **Supplementary Table 4**). It is possible that some aspects of spike morphology resulted in reduced sprouting, or that a nutrient in MS-agar helped to stimulate premature germination. Seri-82 showed constitutive LMA at the warm incubation temperature, even though it carries the LMA-suppressing *Rht-B1b* semi-dwarf allele (**Table 2**; **Figure 6**; Tan, 2004). This is likely due to the presence of the 1B/1R translocation from rye that can cause constitutive LMA in semi-dwarf wheat (Farrell et al., 2013; D. Mares, personal comm.). Interestingly, Seri-82 induces LMA at warmer temperatures and showed susceptibility to vivipary at the warmer incubation temperature (**Supplementary Figure 2**; **Supplementary Table 4**), suggesting that there may be shared mechanisms leading to vivipary and LMA at warmer temperatures. Previous work found no overlap in quantitative trait loci (QTL) for PHS and LMA tolerance in the Cranbrook x Halberd population, but lack of genetic linkage might have been due to limitations in population genetics or marker density (Mrva and Mares, 2001a; Mares and Mrva, 2001). Moreover, it has been suggested that α-amylase regulation during LMA and PHS might share genetic and molecular mechanisms (Derkx and Mares, 2020). Future work should specifically examine whether any shared genetic mechanisms contribute to LMA, PHS, and vivipary tolerance in more diverse germplasm.

The observation that germination could occur during an LMA induction experiment raised interesting questions about whether LMA and premature germination are related phenomena. The current paradigm is that LMA is an inconsistency in α-amylase expression during development that is exacerbated by cool temperature stress during grain maturation (Liu et al., 2021a; Liu et al., 2021b; Mares et al., 2022). It is unclear, however, whether LMA is truly a “defect” or whether it might have some functional benefit to grasses growing in their native environment (Cannon et al., 2022). Since reducing relative humidity can allow LMA induction without visible germination (**Table 2**; **Supplementary Table 4**), it is possible that LMA is indirectly related to premature germination in the strict sense of embryo growth and emergence from covering tissues. However, the observation that the cool temperatures used to induce LMA could stimulate visible germination of immature grain raised the possibility that LMA is a form of vivipary where the germination-related process of α-amylase expression is induced, but the grain doesn’t proceed all the way to visible germination. If true, this would suggest that α-amylase expression occurs during LMA to prepare the grain for germination/vivipary. This would resemble “incipient sprout” in instances where α-amylase is expressed prior to visible mature grain germination as a consequence of sprout-inducing rain (Joe et al., 2005). Future work may better tease apart the regulation of α-amylase by moisture and cool temperatures.

The expectation was that if vivipary was better related to PHS than LMA, then there would be much higher levels of α-amylase closer to the embryo because during mature grain germination GA from the embryo induces α-amylase expression (Mrva and Mares, 1996; Mrva et al., 2006). This was examined by determining α-amylase levels in half-kernels of wheat where vivipary was induced by plating on MS-agar (**Figure 6**) or in detached wheat spikes exposed to cool, humid conditions (**Figure 7**). Interestingly, the ratios of α-amylase at the embryo-proximal half to embryo-distal-half of the kernel were more similar in vivipary and LMA than in vivipary and PHS (**Figure 7**; **Supplementary Figure 6**). It is possible that a germinating grain that is not yet quiescent and is still attached to the mother plant does not need to mobilize starch from the endosperm in the same way as a fully mature/quiescent grain free of the mother plant. Grain at the soft dough stage of maturation is still undergoing grain filling, and the carbohydrate for grain filling comes from the mother plant (reviewed by Bewley et al., 2013). It is unclear what the role of α-amylase induction is during grain filling, in LMA or vivipary, but the source-sink relationships clearly differ from the mature seed. Future work may further address this by examining whether grains undergoing vivipary express PHS-specific transcripts or enzymes. Previous work showed that *TaAmy1* is expressed during PHS and LMA, but *TaAmy2* is expressed only during PHS (Barrero et al., 2013). Moreover, α-glucosidase enzyme activity was detected during PHS, but not during LMA (Mangan et al., 2022). Thus far, there is no molecular marker known to be present during LMA but not PHS.

The fact that cooler temperatures stimulate LMA, PHS, and vivipary raises the question as to whether similar hormonal mechanisms may be involved. Cold stratification (cool, wet conditions) breaks dormancy in mature grain, especially if the grain is thereafter shifted to a warmer temperature (Tuttle et al., 2015). This dormancy-breaking treatment is associated with increasing sensitivity to the germination-promoting hormone GA, as well as with a decrease in ABA levels and sensitivity in wheat. ABA accumulation during grain maturation is responsible both for establishing dormancy and for preventing premature germination (reviewed by Bewley et al., 2013). Thus, the regulation of ABA accumulation during grain maturation may impact both mature and premature grain germination. GA and ABA have also been implicated in stimulating and inhibiting LMA in wheat, however contradictions exist. The GA-insensitive *Rht-D1b* and *Rht-B1b* alleles are associated with lower LMA susceptibility (reviewed by Mares and Mrva, 2014). Application of ABA and GA to LMA-susceptible wheat spikes appeared to be associated with decreased and increased α-amylase levels, respectively (Kondhare et al., 2012; Kondhare et al., 2013; Kondhare et al., 2015). Although the candidate gene for the LMA QTL on chromosome 7B encodes a GA biosynthesis gene homologue, the susceptible allele does not appear to be associated with increased GA levels (Derkx et al., 2021; Mares et al., 2022). Previous work showed that increasing GA levels during grain maturation are associated with vivipary in maize and that GA deficient mutations suppress vivipary in ABA-deficient maize (White et al., 2000). This suggests that stimulation of vivipary by cooler temperatures in wheat may result from increasing GA levels (**Figures 2**, **3**; **Supplementary Figure 4**). Future work will need to examine if wheat vivipary is associated with changes in GA and ABA hormone content and sensitivity.

The current study suggests that LMA and vivipary may result from similar mechanisms in terms of α-amylase regulation and localization but does not provide clear evidence as to whether susceptibility results from the same genetic loci. If vivipary and LMA result from different genetic mechanisms, then it will be important to develop selection methods and molecular markers for each of the two phenomena for use in breeding. In the current study, use of a dehumidifier to keep relative humidity below 75% prevented visible sprouting on the mother plant (**Table 1**), but did not fully prevent germination of detached tillers (**Supplementary Table 4**). We observed a higher propensity for vivipary when LMA was induced using the detached tiller method (**Table 1**; **Supplementary Table 4**) than when LMA was induced on the mother plant (**Tables 1C**, **2**), likely because the detached tiller design uses open buckets of water which can lead to splashes or increase the humidity in a closed chamber environment (Mrva and Mares, 2001b; Liu et al., 2021b). Recording and/or controlling humidity during LMA induction experiments will be important to confidently determine whether susceptibility to LMA and vivipary result from the same or different genetic loci.

Low falling numbers can occur due to pre-harvest sprouting and late maturity α-amylase. This study suggests that vivipary can also cause low FN in the field, because vivipary was significantly associated with low FN in a winter association mapping population and was associated with increased α-amylase expression in spring wheat (**Figures 1**, **7**; **Supplementary Table 4**). It is important to be able to develop wheat varieties that have a lower risk of low falling numbers because of the discounts that farmers receive when selling their grain due to this quality issue. Our research has documented that vivipary can also occur in wheat, causing α-amylase problems. Resistance to vivipary may be another factor to consider when selecting wheat varieties for higher falling numbers. Knowledge about precocious germination can help inform decisions about variety choice and irrigation management.

## Data availability statement

The original contributions presented in the study are included in the article/**Supplementary Material**. Further inquiries can be directed to the corresponding author.

## Author contributions

SP, MP, and CM designed the experiments. MW conducted the experiment in **Figure 2**. The experiments in **Figures 7**, **8** were performed by SP and SC. The remaining experiments were performed by SP. SP and SC performed statistical analyses. SP and CS wrote the manuscript. SP, SC, CS, and MP edited the manuscript. All authors contributed to the article and approved the submitted version.
