# Supplementary material for: LMA or vivipary? Wheat grain can germinate precociously during grain maturation under the cool conditions used to induce late maturity alpha-amylase (LMA)
Source: Front Plant Sci. 2023 Jun 29;14:1156784. doi: 10.3389/fpls.2023.1156784 (PMC10338928; doi:10.3389/fpls.2023.1156784)
Supplement: Supplementary file 1 [file DataSheet_1.pdf]

Supplementary Material

LMA or vivipary? Wheat grain can germinate precociously during grain maturation under the cool conditions used to induce late maturity alpha-amylase (LMA)

Sarah R. Peery, Scott W. Carle, Matthew Wysock, Michael O. Pumphrey, Camille M. Steber*

*** Correspondence:** Camille M. Steber: Camille.steber@usda.gov

1 Supplementary Figures

A

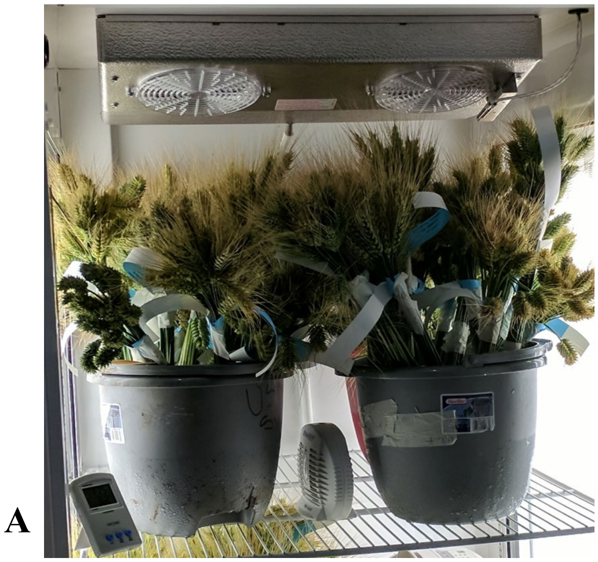

B

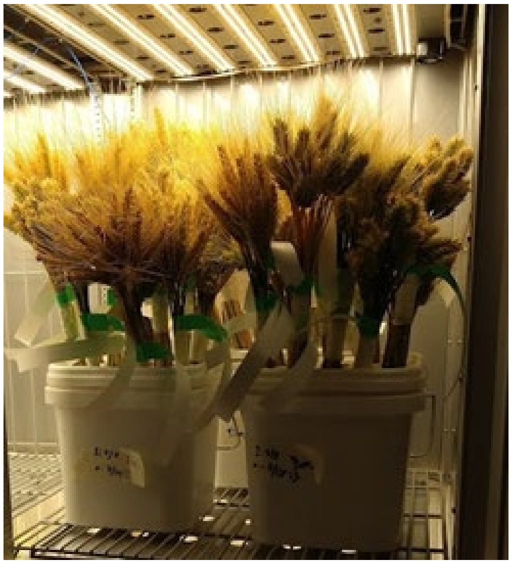

Supplementary Figure 1. Improved bucket design for LMA induction. **(A)** is the previous bucket design and **(B)** is the improved bucket design with holes for each bouquet to increase air circulation around the spikes, reducing moisture condensation.

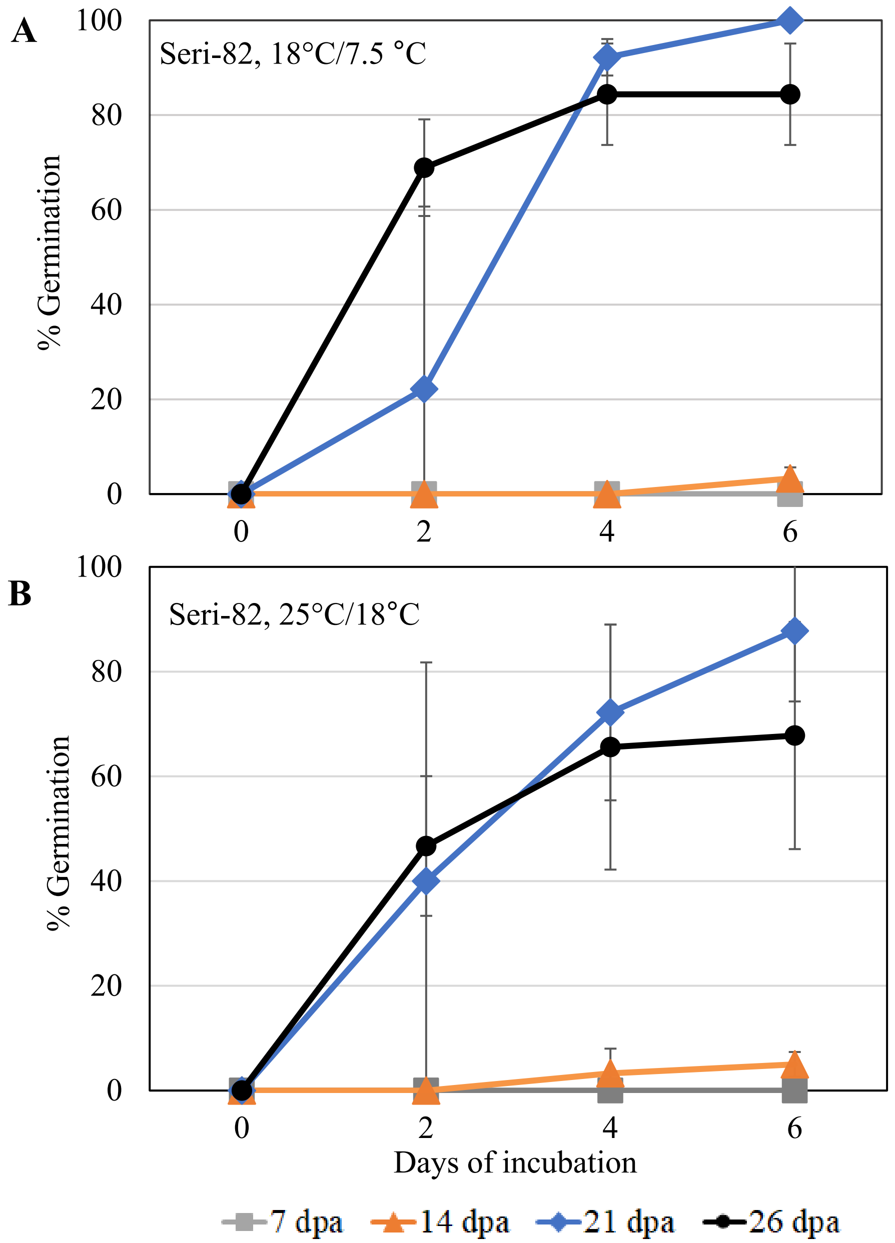

Supplementary Figure 2. Vivipary time course experiment used to determine when Seri-82 was able to germinate before maturity. The Seri-82 plants were grown under warm conditions, 25°C day/18°C night, grain was harvested at the indicated number of days past anthesis (dpa), plated on MS-agar, and incubated at the warm temperature 25°C day/18°C night **(A)** and cool temperature 18°C day/7.5°C night **(B)** as described in Figure 2. Three replications/spikes per treatment were plated with 30 grains from one spike per plate.

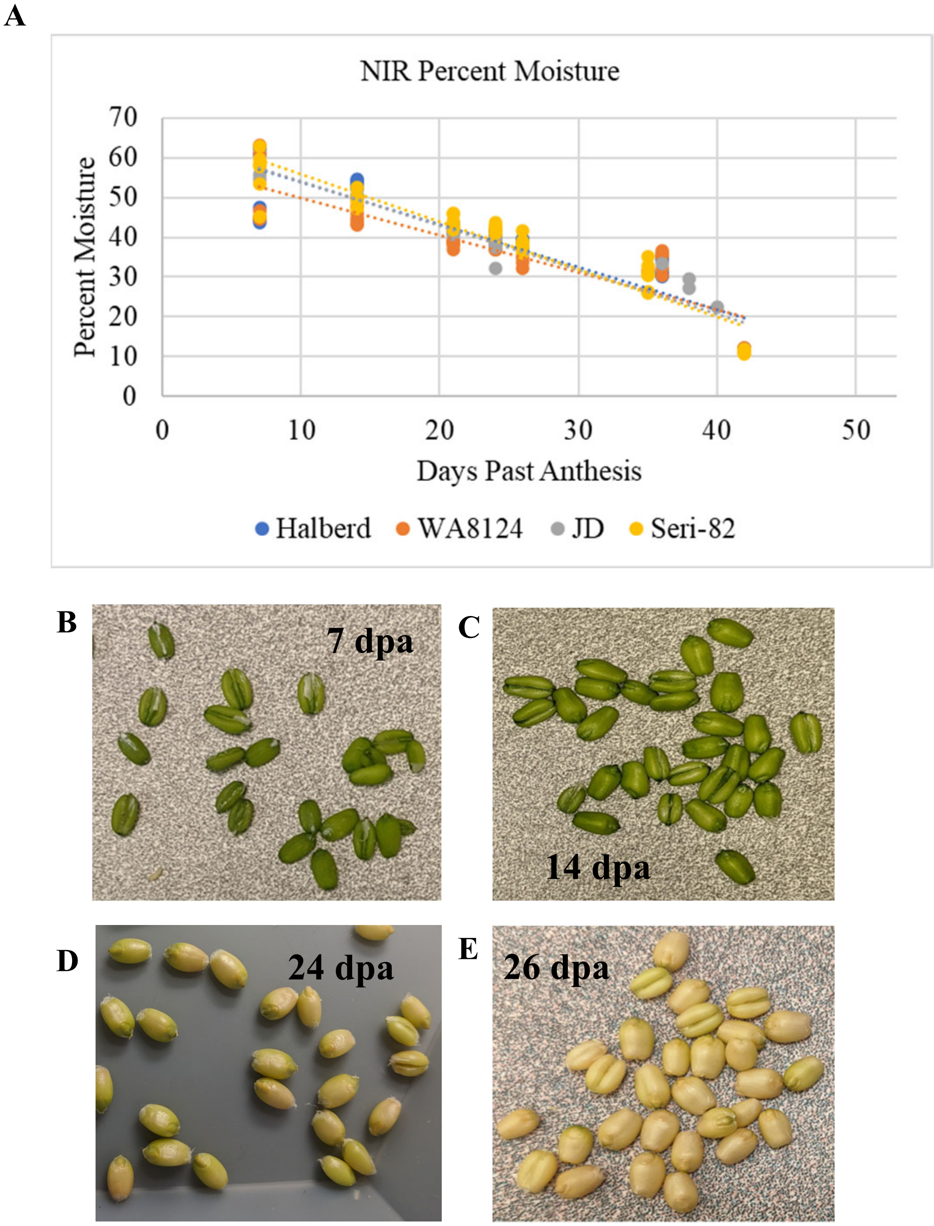

Supplementary Figure 3. Percent Moisture Data for the Developmental Time Course **(A)** Percent grain moisture was obtained using the Results Plus™ software on the Perten NIR 7250 machine for Halberd, WA8124, JD, and Seri-82. Plants were grown at 25°C day/18°C night. Spikes were collected and grains dissected from immature spikes at the indicated number of days past anthesis. **(B, C)** Grains at 7 and 14 dpa appeared to be at the milk stage of grain maturation. **(D, E)** Grains at 21-26 dpa appeared to be at the early to late soft dough stage. During the soft dough stage grain moisture was 35-45%. Grains at the 28 and 30 dpa timepoints appeared to be at the early hard dough stage. Grain collected at 35-40 dpa were at physiological maturity, and grain collected at 42 dpa were at harvest maturity. Trendlines are based on linear regression, Halberd ($y = -1.07 + 64.66$, $r^2 = 0.81$) , WA8124 ($y = -0.94x + 59.39$, $r^2 = 0.77$), JD ($y = -1.12x + 65.40$, $r^2 = 0.90$), Seri-82 ($y = -1.20x + 67.92$, $r^2 = 0.90$).

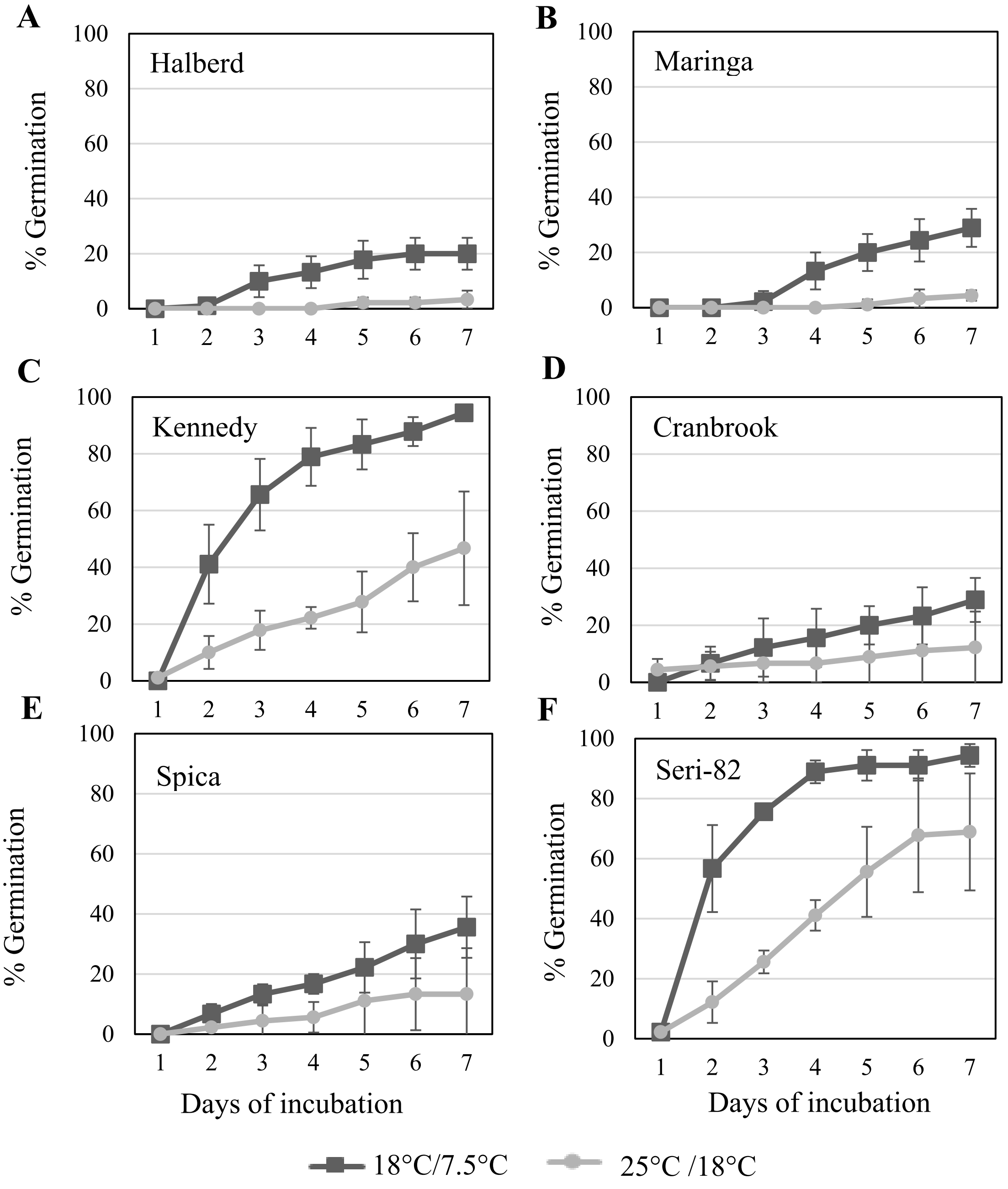

Supplementary Figure 4. Vivipary germination assays showed higher germination at the cooler incubation temperature. Grain was removed from spikes at 24-26 dpa, plated on MS-agar and incubated at either the warm (grey) 25°C day/18°C night or cool (black) 18°C day/7.5°C night treatment temperature. Mean percent germination of 12 replications of 30 grains per temperature per genotype is shown. Error bars represent SD The cooler incubation temperature significantly increased premature germination (*p*-value = 0.006, Student's t-test) Error bars show SE.

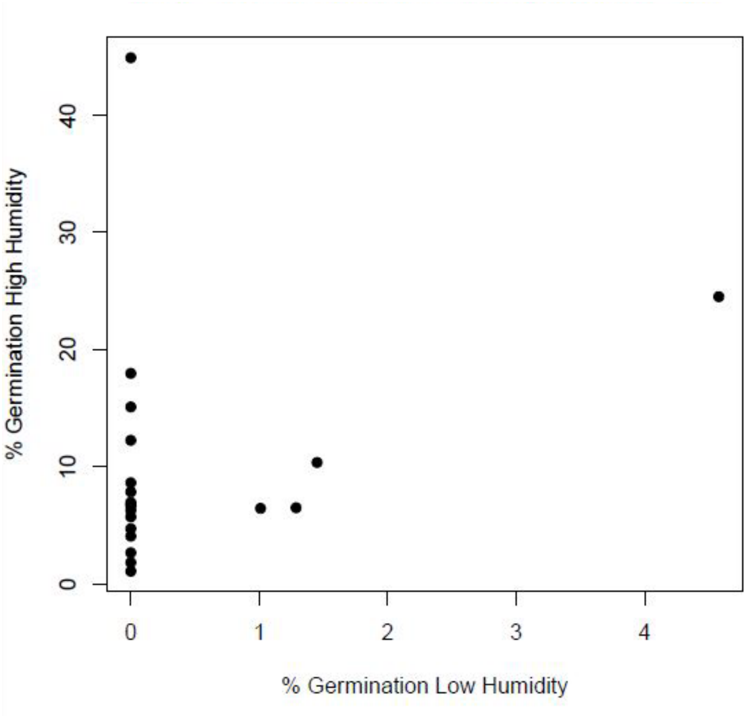

Supplementary Figure 5. Comparison of the percent germination of kernels obtained from spikes cool-treated at high versus low relative humidity. The percent germination of all field replications were averaged by plot after treatment at high or low humidity. This resulted in a total of 40 datapoints using combined data from the eight genotypes, Halberd, Kennedy, Cranbrook, JD, WA8124, Maringa, Seri-82, and Spica. Note that the y- and x-axis scales are different.

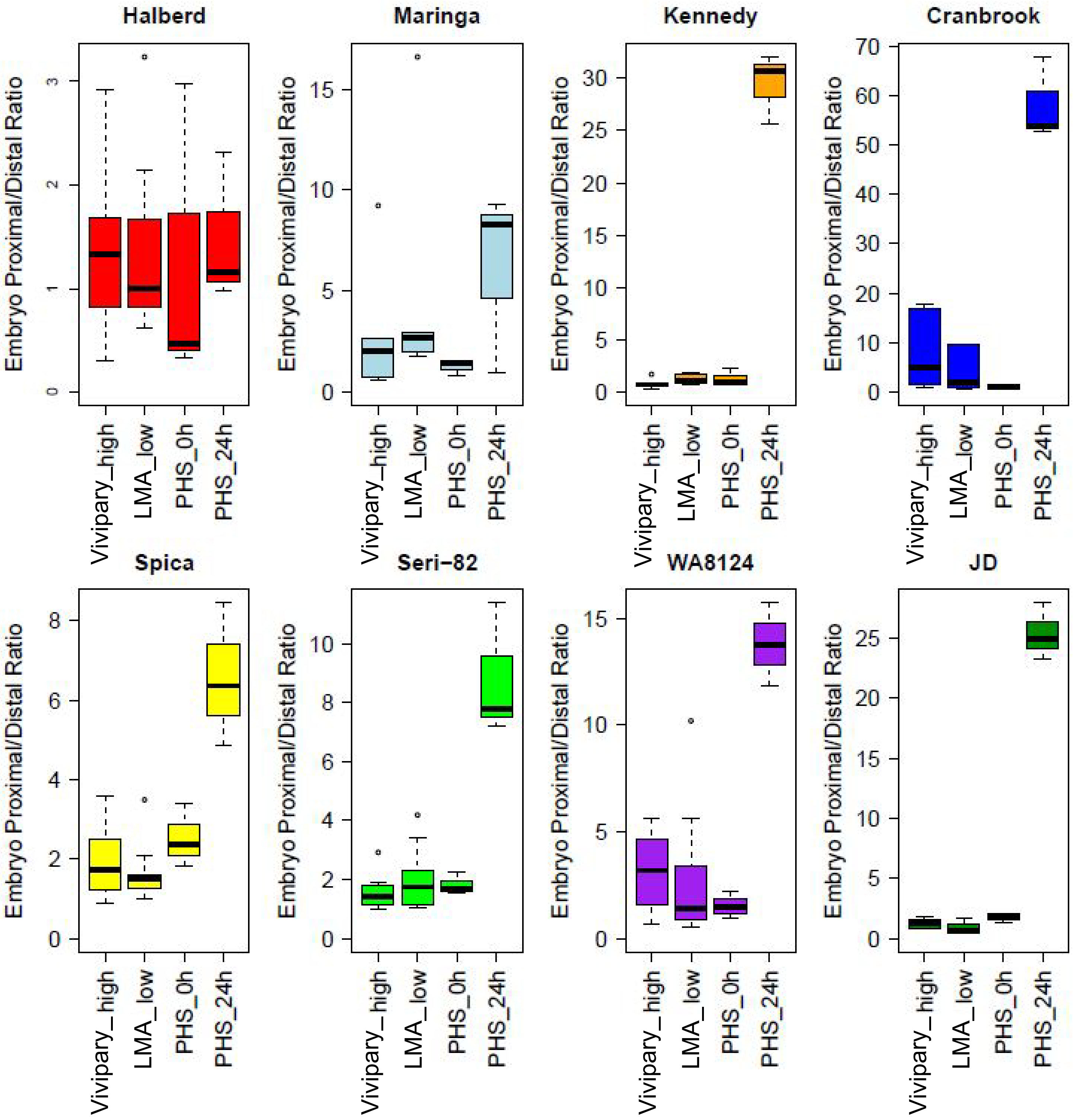

Supplementary Figure 6. The alpha-amylase ratio during vivipary better resembles LMA than PHS. Comparison of embryo-proximal to embryo-distal alpha-amylase activity ratios in the high (vivipary) and low (LMA) humidity experiments to mature seed germination (PHS 24hr). The ratio of embryo-proximal to -distal half-kernel alpha-amylase enzyme activity (Au) are shown on the y-axis from spikes that were incubated at 18°C day/7.5°C night at either high or low relative humidity as given in Figure 7. Note that the y-axis scale varies between plots. These values are compared to half-kernel ratios from mature grain that was imbibed for 0 hr and 24 hr at 22°C as in Figure 6. Error bars indicate SE.

2 Supplementary Tables

Supplementary Table 1. Visible sprouting scale used to score spike-wetting assays.

Score	Seminal roots		Coleoptiles	
	Number	Length	Number	Length
1			No visible sprouting	
2	1-2 per spike	Just emerging	-	-
3	3-4 per spike	1-2 mm	-	-
4	65-75% of the spikelets	3-4 mm	-	-
5	Uniform over the spike	4-6 mm	-	-
6	Uniform over the spike	6mm – 1cm	1 or 2 per spike	Just emerging
7	Uniform over the spike	1-2 cm	>2 per spikes	Just emerging
8	Uniform over the spike	2-4 cm	Uniformly emerging	0-0.5 cm
9	Uniform over the spike	>4 cm	Uniform over the spike	1-2 cm
10	Uniform over the spike	>4 cm	Uniform over the spike, first leaf emerging	3-4 cm

Supplementary Table 2. ANOVA for the 2017 QAM LMA induction experiment

A. ANOVA of the 2017 QAM, the effect of cool treatment on FN

	DF	SUM Sq	Mean Sq	F value	Pr(>F)[a]
Treatment effect	1	0.435	0.4354	8.771	0.00322*
Residuals	449	22.287	0.0496		

B. ANOVA of the 2017 QAM, the effect of low FN on visible sprouting**.**

	DF	SUM Sq	Mean Sq	F value	Pr(>F)[b]
FN effect	1	15.58	15.584	74.74	$<2 \times 10^{-16}$***
Residuals	451	94.04	0.209		

[a]$p < 0.01$*.

[b]$p < 0.0001$***.

Supplementary Table 3. ANOVA comparing factors influencing LMA inductions

	DF	SUM Sq	Mean Sq	F value	Pr(>F)[d]
Experiment[a]	1	1.82	1.82	3.0794	0.08015
Plant[b]	1	1.36	1.36	2.2965	0.13056
DPA[c]	1	2.86	2.86	4.8274	0.02866*
Genotype	8	226.32	28.29	47.7788	$<2 \times 10^{-16}$***
Residuals			354	209.606	0.5921

[a]Experiment was repeated three times.
[b]Plants were grown in separate pots.
[c]Days Post Anthesis
[d]$p < 0.01$*, $p < 0.0001$***.

Supplementary Table 4. Percent Germination and alpha-amylase enzyme activity in half kernels after cool treatment at high and low regulative humidity.

Genotype	%G High[a] Mean ± SE	%G, Low[a] Mean ± SE	High[a] P[b] Mean + SE Activity (Au)	High[a] D[b] Mean + SE Activity (Au)	n[c]	Low[a] P[b] Mean + SE Activity (Au)	Low[a] D[b] Mean + SE Activity (Au)
Halberd	8 ± 4.4	0 ± 0.0	0.48 ± 0.40	0.22 ± 0.13	9	0.08 ± 0.01	0.06 ± 0.01
Maringa	12 ± 4.3	0 ± 0.0	1.12 ± 0.48	0.46 ± 0.09	6	1.27 ± 0.59	0.29 ± 0.04
Kennedy	5 ± 2.9	0 ± 0.0	0.14 ± 0.04	0.19 ± 0.06	6	0.18 ± 0.03	0.15 ± 0.03
Cranbrook	8 ± 2.3	2 ± 0.8	2.30 ± 0.62	0.63 ± 0.36	6	1.63 ± 0.64	0.44 ± 0.12
Spica	18 ± 10.5	0 ± 0.0	2.46 ± 0.35	1.54 ± 0.36	9	2.78 ± 0.38	1.95 ± 0.43
Seri-82	15 ± 5.1	3 ± 0.8	2.87 ± 0.38	2.08 ± 0.38	9	3.15 ± 0.25	1.83 ± 0.25
JD	6 ± 3.0	0 ± 0.0	0.30 ± 0.06	0.25 ± 0.05	6	0.31 ± 0.11	0.38 ± 0.11
WA8124	7 ± 2.1	1 ± 0.3	2.18 ± 0.48	0.49 ± 0.15	9	0.81 ± 0.20	0.62 ± 0.05
WA8148	7 ± 1.4	0 ± 0.0	--	--	9	--	--
HR07024-5	46 ± 16.2	0 ± 0.0	--	--	6	--	--
Babe	24 ± 23.5	1 ± 1.2	--	--	3	--	--
UC1599	7 ± 3.3	1 ± 0.5	--	--	9	--	--
AC-Cadillac	13 ± 4.5	0 ± 0.0	--	--	6	--	--
Waskada	23 ± 9.2	0 ± 0.0	--	--	6	--	--
MN06075-4	18 ± 1.2	0 ± 0.0	--	--	9	--	--

[a]High and Low indicate that the cool treatment at 18°C day/7. 5°C night was conducted at a high 54-95% relative humidity or a low 40-75% relative humidity.
[b]P indicates the embryo proximal half-kernel alpha-amylase enzyme activity and D indicates that embryo distal half-kernel enzyme activity.
[c]n is the number of spikes examined for percent germination and the number of spikes sampled for half-kernel assays.
